# Genome-Wide Association Reveals Signalling-Linked Infection Tolerance in Hibernating Bats

**DOI:** 10.3390/pathogens15020149

**Published:** 2026-01-30

**Authors:** Markéta Harazim, Lubomír Piálek, Hana Bandouchova, Jiri Pikula, Veronika Seidlová, Jan Zukal, Monika Němcová, Tomas Heger, Petr Linhart, Vladimír Piaček, Tomasz Kokurewicz, Oleg L. Orlov, Alexandra Zahradníková, Natália Martínková

**Affiliations:** 1 Institute of Vertebrate Biology, Czech Academy of Sciences, Květná 8, 60300 Brno, Czech Republiczukal@ivb.cz (J.Z.); 2 Department of Botany and Zoology, Faculty of Sciences, Masaryk University, Kotlářská 2, 61137 Brno, Czech Republic; 3 Department of Zoology, University of South Bohemia in České Budějovice, Branišovská 31, 37005 České Budějovice, Czech Republic; 4 Avian and Exotic Animal Clinic, Faculty of Veterinary Medicine, University of Veterinary Sciences Brno, Palackého třída 1946/1, 61242 Brno, Czech Republic; bandouchovah@vfu.cz; 5 Department of Ecology and Diseases of Zoo Animals, Game, Fish and Bees, University of Veterinary Sciences Brno, Palackého třída 1946/1, 61242 Brno, Czech Republic; pikulaj@vfu.cz (J.P.); seidlovav@vfu.cz (V.S.); nemcovam@vfu.cz (M.N.); hegert@vfu.cz (T.H.); piacekv@vfu.cz (V.P.); 6 Department of Animal Protection and Welfare & Veterinary Public Health, University of Veterinary Sciences Brno, Palackého třída 1946/1, 61242 Brno, Czech Republic; linhartp@vfu.cz; 7 Institute of Biology, Department of Vertebrate Ecology and Paleontology, Wrocław University of Environmental and Life Sciences, 51-631 Wrocław, Poland; tomasz.kokurewicz@upwr.edu.pl; 8 Department of Biochemistry, Tyumen State Medical University, Odesskaya St. 54, 625023 Tyumen, Russia; o_l_orlov@mail.ru; 9 Department of Cellular Cardiology, Institute of Experimental Endocrinology, Biomedical Research Center of the Slovak Academy of Sciences, Dubravska cesta 9, 84545 Bratislava, Slovakia; saschia.sk@gmail.com; 10 RECETOX, Faculty of Sciences, Masaryk University, Kotlářská 2, 61137 Brno, Czech Republic

**Keywords:** Chiroptera, white-nose syndrome, genome-wide association study, adaptation, infection

## Abstract

Hibernation profoundly alters host–pathogen dynamics by suppressing metabolism and immune function, posing unique challenges for infection control. In this study, we examined how genomic variation modulates infection and physiological traits in temperate bats during hibernation. We combined infection screening, haematology, blood biochemistry, and whole-genome sequencing across five vespertilionid species, identifying over 170,000 single nucleotide variants (SNVs) and assessing their associations with 23 health-related variables. Using the phylogenetically informed treeWAS framework, we detected 515 significant SNVs linked to traits including fungal, protozoan and bacterial infections, acid–base balance, and blood cell indices. These SNVs mapped to 137 unique genes, which were enriched for functional domains related to cytoskeletal dynamics, membrane trafficking, and intracellular signalling (e.g., SH3, C2, BAR, semaphorin). Notably, canonical immune effector genes were underrepresented, and several trait-associated SNVs appeared in blocks across multiple scaffolds, pointing to regulatory loci as key modulators of hibernator health. Our findings support the hypothesis that bats rely on infection tolerance rather than resistance during hibernation, with genomic variation in regulatory and signalling pathways shaping their physiological responses to infection under energy-limited conditions.

## 1. Introduction

Bats are exceptional among mammals for their longevity, ecological diversity, and capacity to carry numerous pathogens without developing clinical disease [1,2,3]. Despite frequent exposure to viruses, bacteria, protozoa, and fungi, they often survive infections that are lethal in other taxa such as rabies or Ebola [2]. This has raised interest in their role as natural reservoirs of zoonotic pathogens and in the mechanisms underlying their infection tolerance [4,5]. Their distinct physiological and ecological traits, including flight, heterothermy, specific roosting behaviour, and long-distance migration are thought to influence both pathogen exposure and immune function [6,7,8,9].

In temperate regions, many bat species undergo hibernation, marked by reduced body temperature, metabolic rate, and immune activity [10,11]. These physiological changes alter host–pathogen dynamics in multiple ways, including suppression of inflammatory responses, modulation of pathogen replication, and changes in transmission dynamics. Hibernation suppresses inflammation and limits leukocyte circulation, reducing typical immune responses [11,12,13,14]. At the same time, it can inhibit replication of temperature-sensitive cellular pathogens and viruses dependent on host cellular activity for replication, potentially lessening infection severity [15,16,17,18]. In contrast, the aggregation of hibernating individuals may enhance pathogen transmission, with effects varying with roosting behaviour [19,20,21].

These dynamics are especially relevant for fungal pathogens adapted to cold conditions. The causative agent of white-nose syndrome, *Pseudogymnoascus destructans*, has been detected in multiple bat species across the Palearctic [22,23], but generally without associated population declines [23]. The fungus proliferates during torpor at low body temperatures while host immune activity is suppressed, leading to progressive tissue damage that accumulates across torpor bouts and is exacerbated by energetically costly arousals [14,15,24,25,26]. Disease progression therefore reflects a mismatch between pathogen growth during metabolic suppression and energetically costly immune activation during arousal, making hibernation a natural model for studying how host physiology constrains infection outcomes under extreme metabolic conditions.

Variability in the physiological response may reflect genetic factors shaping individual and species-level responses to infection, acting through immune recognition, inflammatory control, and host tissue resilience. Bats show signatures of accelerated evolution in immune-related genes, including those involved in viral detection and regulation of inflammation [12,27]. Variation in genes such as the major histocompatibility complex, interferon-stimulated genes, and Toll-like receptors contributes to inter-individual and interspecific differences in infection susceptibility and tolerance [27,28,29]. Beyond canonical immune system genes, adaptations in pathways maintaining primary barrier integrity and tissue repair may enhance survival during infection under the extreme conditions of hibernation [30].

Understanding how ecological traits, physiological states, and genetic variation together influence disease dynamics is essential for both disease ecology and bat conservation [3,17,31]. In this study, we aim to identify genetic factors that modulate infection susceptibility and health status in temperate bats. We combine infection screening with haematological profiles and single nucleotide variant (SNV) data to explore associations between host genotype, infection status, and physiological condition. This integrative approach seeks to clarify how genetic variation contributes to bat–pathogen interactions.

## 2. Materials and Methods

### 2.1. Sample Collection

**Figure 1:**
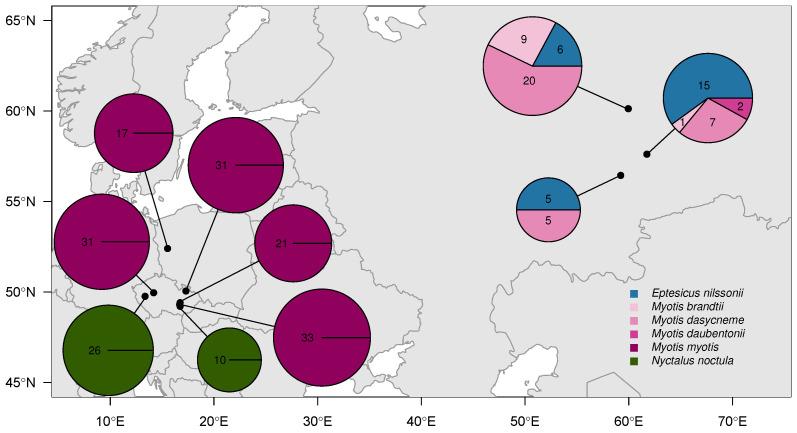
Sampling sites of bats analysed in this study. Black dots indicate site locations. Pie charts represent the relative abundance of each bat species at a given site, with chart size scaled to the total number of individuals sampled. Sample sizes are indicated by numbers in each pie section.

We sampled 261 bats in Czechia, Poland, and Russia between 2016 and 2018 (Figure 1). Bats were located on hibernaculum walls between December and May. We swabbed the surface of the left wing (FLOQ Swabs, Copan Flock Technologies srl, Brescia, Italy) in a standardised manner to collect fungal biomass for quantification of *P. destructans* load [15], and we transilluminated the left wing with ultra-violet (UV) light and photographed it to visualise characteristic orange-yellow lesions associated with white-nose syndrome. We later counted the number of UV fluorescent spots per cm^2^ of wing area as a proxy for tissue damage.

After photography, bats were placed into cotton bags to allow arousal from torpor. Following a re-warming period of approximately 60 min, the skin over the uropatagial vessel was disinfected with alcohol, and a single blood sample (100 μL) was collected using a heparinised capillary tube [32]. The blood sample was immediately used for on-site physiological and biochemical measurements using a portable i-STAT analyser (Abaxis, Union City, CA, USA), and a subsample was used to prepare a blood smear for haematological examination. The remaining blood was used for parasitaemia quantification.

Not all variables were measured in all individuals, reflecting variation in logistical conditions and sampling objectives across sites and time points. Haematological and biochemical parameters were successfully obtained for subsets of the full dataset, with sample sizes ranging from 16 individuals (for complete blood counts) to over 90 (for selected blood chemistry markers). While analyses are necessarily limited to individuals with complete data for each variable, these subsets remain suitable for assessing associations within defined contexts.

Before release at the hibernaculum, bats were supplemented with fluids and energy by oral administration of a glucose and saline solution. All handling was performed by qualified personnel to minimise distress, and blood sampling was conducted in accordance with the Animal Ethics Procedures and Guidelines of the University of Veterinary Sciences Brno, Czechia.

### 2.2. Haematological Parameters

We treated the blood smears with the Romanowsky stain. We then determined differential white blood cell counts by counting 100 leukocytes under oil immersion magnification and we calculated the relative number of lymphocytes, monocytes, neutrophils, basophils and eosinophils.

Physiological and biochemical parameters were measured on site using an i-STAT portable analyser with EC8+ cartridges (Abaxis). The analysed variables included haematocrit (Hct), haemoglobin (Hb), sodium (Na), potassium (K), chloride (Cl), total carbon dioxide (tCO_2_), partial pressure of carbon dioxide (pCO_2_), bicarbonate (HCO_3_), base excess (BE), anion gap (AnGap), blood pH, glucose, and urea.

These parameters provide a comprehensive overview of haematological condition, acid–base balance, and metabolic state in hibernating bats. Reference intervals for comparable *Myotis* species are provided by Bandouchova et al. [33].

### 2.3. Identification and Quantification of Pathogens

We isolated fungal DNA from wing swabs using the QIAamp DNA Mini Kit (Qiagen, Halden, Germany) and quantified fungal load with quantitative PCR (qPCR). To detect *P. destructans*, we applied a dual-probe TaqMan assay (Life Technologies, Foster City, CA, USA) following the protocol of Zukal et al. [22], which included triplicate samples, positive and negative controls, and a calibration curve based on a dilution series of a positive control. Fungal load was expressed as log_10_(1 + ng cm^−2^) to allow inclusion of zero values. The number of UV fluorescent lesions was calculated as log_10_(1 + number cm^−2^) based on photographs taken during UV transillumination of the dorsal side of the left wing.

Infection status for *Trypanosoma* and *Bartonella* was determined using a combination of microscopy and nested PCR following the protocols of Schorn et al. [34] and Linhart et al. [35]. Blood smears were screened under light microscopy to assess the presence of trypomastigotes for *Trypanosoma*. To confirm these findings and detect low-level infections, we extracted DNA from whole blood samples and applied nested PCR targeting to the 18S rDNA gene for *Trypanosoma dionisii* and *Trypanosoma vespertilionis* [35]. A subset of PCR-positive samples was sequenced for taxonomic confirmation, and these sequences have been published previously [35]. For *Bartonella*, we applied a PCR assay targeting the 16S rRNA [34]. For the purposes of this study, infection status was recorded as a binary variable (infected/uninfected) based on combined evidence from microscopy and PCR for *Trypanosoma*, and PCR alone for *Bartonella*.

### 2.4. DNA Purification and Library Preparation

We preserved tissue samples in 70% ethanol and extracted genomic DNA using the Exgene™ Tissue SV kit (GeneAll Biotechnology Co., Ltd., Seoul, Republic of Korea), following the manufacturer’s protocol. To construct two ddRAD libraries, we followed the protocol of Piálek et al. [36]. We digested DNA with the restriction enzymes SphI-HF^®^ and MluCI (New England BioLabs, Ipswich, MA, USA), and ligated unique barcodes to both the 5′ and 3′ ends of each sample. We selected DNA fragments between 276 and 324 bp using the Pippin Prep™ platform (Sage Science, Inc., Beverly, MA, USA). Library sequencing was performed on an Illumina HiSeq4000 platform with 150 bp read length by a commercial provider.

### 2.5. Sequencing Data Processing

We processed the two sequencing runs separately using process_radtags in Stacks v2.3e [37], demultiplexing reads based on inline barcodes and removing low-quality reads, adapter contamination, and Illumina artefacts. Cleaned reads were mapped individually to the *Myotis myotis* reference genome (GCF_014108235.1) using Bowtie2 v2.2.4 with the –very-sensitive-local preset [38]. We converted and sorted the resulting SAM files to BAM format using Samtools v1.3.1 [39].

Since all ddRAD reads were mapped to the *M. myotis* reference genome, mapping efficiency and sequencing depth may vary among genera due to sequence divergence from the reference. To assess the reproducibility of library construction and the uniformity of sequencing depth, we quantified per-sample sequencing depth at all SNV loci included in the treeWAS analysis using Samtools. For each sample, we summarised depth using (1) locus callability metrics, defined as the proportion of loci exceeding minimum depth thresholds (DP ≥ 5, DP ≥ 10, and DP ≥ 20) and the proportion of loci with zero coverage (DP = 0), and (2) descriptive depth statistics, including mean, standard deviation, median, and the median sequencing depth conditional on loci with non-zero coverage (DP > 0). The conditional median depth provides a robust summary of sequencing depth in the presence of locus dropout typical of reduced-representation sequencing. Depth distributions were compared between the two libraries in R v4.5.2 [40] to evaluate technical consistency.

We called variants using bcftools v1.14 [39]. First, we generated variant likelihoods using bcftools mpileup with allelic depth annotation and multithreaded processing. We then called variants using the multiallelic caller (bcftools call -mv) and retained only variant sites. To improve data quality, we filtered variants to retain only those with a minimum allele frequency greater than 0.05 using bcftools filter. Finally, we excluded individuals with more than 50% missing data using vcftools [41]. This resulted in a high-quality SNV dataset for downstream analysis.

### 2.6. Reconstruction of Relatedness Structure

Dependencies between samples influence statistical estimates of genomic associations with traits. To correct for the relatedness structure in our data, we employed two approaches: a phylogenetic reconstruction and genome polarisation.

We reconstructed the phylogeny from the SNV matrix using a maximum likelihood (ML) framework in IQ-TREE v2 [42]. We applied a GTR + Γ substitution model with ascertainment bias correction to account for the fact that SNV matrices include only variant sites. Node support was assessed using 1000 ultrafast bootstrap replicates, and nodes with support ≥95% were considered robust. We verified the ML phylogeny using Bayesian inference to confirm major clades.

We then used genome polarisation [43] to estimate genomic admixture between our taxa. While the species included in this study are not known to frequently hybridise, genome polarisation can reveal subtle patterns of shared genomic variation [44]. Importantly, this approach does not assume prior knowledge of marker diagnosticity or sample assignment. Instead, it infers both from genome-wide associations between marker states across individuals, allowing unbiased identification of informative genetic structure. Genome polarisation using the *diem* algorithm in the diemr v1.5.2 R package [40,43] reorients genotypes across individuals by identifying alleles that are informative for distinguishing species or lineages. First, the VCF file was converted to *diem* format, requiring that each SNV had at least one homozygous individual for each of the two most frequent alleles at the site. We then ran the *diem* algorithm on all retained sites to estimate the polarity and diagnostic index of each site. Finally, we visualised polarised genotypes for the 20% of sites with the highest diagnostic index. This approach highlights sites that are informative for distinguishing species while reducing the confounding influence of shared ancestral polymorphism and standing genetic variation [45].

### 2.7. Genome-Wide Association Study

We conducted a phylogenetically informed genome-wide association study using the R package treeWAS v1.0 [46]. SNVs were extracted from a filtered and recoded VCF file using the vcfR v1.15.0 package [47]. Indels and heterozygous genotypes were removed, and biallelic SNVs were recoded into binary presence–absence format for each allele. Individuals with more than 50% missing data were excluded. A maximum likelihood phylogeny, constructed from the same SNV dataset using IQ-TREE (see above), was pruned to match the sample set. We tested associations between SNVs and infection and health variables (Table 1) using the uncorrected *p*-value threshold of $10^{-5}$. For each trait, we ran the default treeWAS pipeline and recorded results for the terminal, simultaneous, and subsequent association scores. The terminal association score evaluates the correlation between the presence of an SNV and a trait at the tips of the phylogeny, detecting lineage-specific associations. The simultaneous score accounts for both the phylogenetic structure and the distribution of the trait and SNV across the tree, identifying associations that may have arisen through shared ancestry or convergent evolution. The subsequent score captures whether changes in SNV states are consistently followed by changes in trait states along branches of the phylogeny, suggesting a potential causal relationship. The *p*-values are obtained through permutation tests that evaluate how often a score as extreme as the observed one arises under a null distribution that preserves the phylogenetic structure. While correction for multiple testing is typically necessary, treeWAS applies conservative thresholds due to the inherent phylogenetic dependency among SNVs, reducing the risk of false positives even without formal correction [46,48].

**Table 1:** Summary of infection markers and physiological parameters included in association tests. For each variable, sample size is shown; for infection markers, the number of positive samples is given in parentheses. Observed ranges refer to raw data values. All continuous variables were min–max scaled prior to association testing, and their distributional properties (median, interquartile range, and outliers) are shown in Figure 2.

Variable		*n*	Minimum	Maximum	Units
Infection markers					
	*Trypanosoma*	107 (19)	0	1	uninfected/infected
	*Bartonella*	216 (200)	0	1	uninfected/infected
	Fungal load	210 (124)	0	0.24	log_10_(1 + ng cm^−2^)
	UV lesions	85 (33)	0	0.90	log_10_(1 + no. cm^−2^)
Haematological parameters					
	Trombocyte count (PLT)	16	244	2952	10^9^ L^−1^
	Mean corpuscular haemoglobin in RBC (MCH)	16	64.8	100.4	pg
	MCH concentration (MCHC)	15	1364	3124	g dL^−1^
	Mean corpuscular volume of RBC (MCV)	16	168	192	fl
	White blood cell count (WBC)	16	0.8	2.8	10^9^ L^−1^
	Red blood cell count (RBC)	16	7.08	11.48	10^12^ L^−1^
Biochemical parameters					
	Sodium (Na^+^)	92	127	169	mmol L^−1^
	Potassium (K^+^)	91	2.8	8.8	mmol L^−1^
	Chloride (Cl^−^)	89	97	136	mmol L^−1^
	Total CO_2_ (tCO_2_)	91	14	36	mmol L^−1^
	Urea	90	1	50	mmol L^−1^
	Blood pH	91	7.066	7.462	–
	Partial CO_2_ pressure (pCO_2_)	91	3.63	12.02	kPa
	Bicarbonate (HCO_3_)	91	13.1	34	mmol L^−1^
	Base excess (BE)	91	–17	9	mmol L^−1^
	Anion gap (angap)	87	8	32	mmol L^−1^
	Haemoglobin (hb)	91	122	218	g L^−1^
	Glucose	91	1.3	14.2	mmol L^−1^
	Haematocrit	91	36	64	%

**Figure 2:**
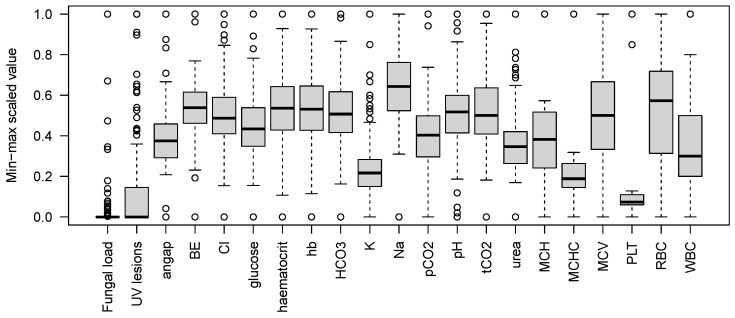
Min-max scaled variation in health and infection parameters across sampled bats. Boxes represent the interquartile range (IQR), with the horizontal line indicating the median; whiskers extend to the most extreme values within 1.5× IQR, and points denote outliers. The figure summarises the distributional properties of scaled variables used in the association analyses.

We identified genes with SNVs significantly associated with health-related variables by intersecting the genomic coordinates of significant SNVs (treeWAS p<0.00005) with the annotated gene models in the *M. myotis* reference genome. Gene symbols and NCBI Gene IDs were extracted for each overlapping gene with bedtools.

To evaluate the functional significance of the affected genes, we used the Database for Annotation, Visualization and Integrated Discovery (DAVID) v6.8 [49,50]. Gene lists were derived from genes containing associated SNVs and submitted as Entrez Gene IDs using the *M. myotis* genome as the background for enrichment analysis. We used the Functional Annotation Clustering tool, which groups enriched terms based on shared genes and functional similarity. For each cluster, DAVID calculates an enrichment score, defined as the negative log_10_ of the geometric mean of the enrichment *p*-values for the terms in the cluster. Clusters with enrichment scores ≥ 1.3 (equivalent to p≤0.05) were considered to have meaningful biological support.

We conducted this analysis both for the full set of genes containing any significantly associated SNV, and separately for genes associated with specific variables, to identify variable-specific functional pathways. However, gene lists associated with individual variables were often short, limiting the statistical power to detect enriched functions.

## 3. Results

### 3.1. Variation in Bat Health Parameters

We observed substantial variation across infection and physiological parameters (Table 1, Figure 2). *Bartonella* infection was detected in 200 of 216 tested individuals, indicating high prevalence (Table 1). In contrast, *Trypanosoma* was found in only 19 of 107 tested bats (Table 1). Fungal load, expressed as log_10_(1 + ng DNA per cm^2^ of wing membrane), ranged up to 0.24, showing variation in pathogen exposure among individuals. The density of UV fluorescent spots on the wing membrane, used as a proxy for tissue damage, spanned nearly an order of magnitude (Table 1).

Health-related traits exhibited wide inter-individual variability. In a subset of 16 individuals with haematological data, thrombocyte counts ranged from 244 to $2952\times 10^{9}$ L^−1^, and white and red blood cell counts, as well as derived indices (MCH, MCHC, MCV), varied accordingly (Table 1). Blood biochemistry parameters (n≈90) showed particularly large ranges for glucose (1.3–14.2 mmol L^−1^), ion concentrations, and acid–base status (Table 1). Together, these data reflect both biological heterogeneity and uneven sampling across traits, which we accounted for in the treeWAS analyses by testing each variable independently in the subset of individuals with available data. Given the presence of outliers and wide measurement ranges, using the rank-based implementation of treeWAS was appropriate to ensure robust inference.

### 3.2. Reproducibility of ddRAD Libraries

Sequencing depth distributions were comparable between the two independently constructed ddRAD libraries (Figure S1). Median sequencing depth of captured loci showed overlapping ranges between libraries across species, with no consistent library-specific shift indicative of technical bias. Although substantial variability in depth was observed among individual samples and species, this variation was not structured by library identity and is consistent with the heterogeneous locus recovery typical of ddRAD data. Samples excluded from downstream analyses generally exhibited lower median depth and greater dispersion but were present in both libraries, supporting the absence of library-specific effects on sequencing depth.

Out of 274 individuals sampled, 239 passed sequencing quality control, mapping, and variant filtering thresholds (Figure S1). The final SNV matrix comprised 174,147 variant sites.

### 3.3. Genomic Divergence

**Figure 3:**
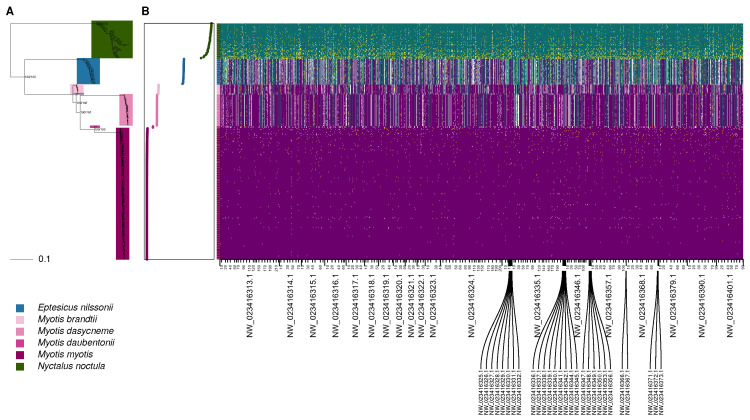
Genomicdivergence between bats. (**A**) Maximum likelihood phylogeny of bat species used in this study. Node labels show ML/BI support at major nodes, and the phylogeny is rooted at midpoint. (**B**) Rescaled hybrid index (left) and genome polarisation plot (right), depicting the 20% of SNVs with the highest diagnostic index. In the genome polarisation plot, individuals are arranged in rows and SNVs in columns. Tick mark colours on the y-axis correspond to species identities, consistent with the hybrid index (left) and the phylogeny (**A**). Tick marks on the x-axis are spaced according to genomic coordinates, with labels indicating scaffold accessions and positions every 10^7^ bp in the reference genome. Purple indicates homozygous genotypes for alleles diagnostic of *Myotis myotis*, green for *Nyctalus noctula*, yellow for heterozygous genotypes, and white for missing data.

The ML phylogeny reconstructed from the SNV matrix revealed distinct separation among all included bat species with high support (Figure 3A). *Nyctalus noctula* was the most divergent taxon, and also showed the greatest intraspecific variance in highly diagnostic sites (Figure 3B). The next lineage to diverge was *E. nilssonii*. The *Myotis* species clustered together as expected, with *M. brandtii* occupying a basal position within the clade. The polarised genomes showed that the 20% sites with the highest diagnostic index are evenly distributed across the scaffolds (Figure 3B). Hybrid indices derived from the polarised genotypes were consistent with the ladderized structure of the phylogeny. We observed very little genetic divergence within *Myotis*, and consistently high homozygosity in diagnostic markers across all species in this genus.

### 3.4. Associations Between Genomic Variation and Health-Related Variables

We identified SNVs significantly associated with health variables across hibernating vespertilionid bats using the treeWAS framework. A total of 515 SNVs surpassed the genome-wide significance threshold (p<0.00005), distributed across 28 scaffolds of the *M. myotis* reference genome (Table S1). Significant associations were observed for 22 out of 23 tested variables, where WBC had no significant associations with the genotyped SNVs in the dataset.

The strongest association signal varied across variables, with most SNVs supported by either the terminal (score.1; 142 SNVs) or simultaneous (score.2; 292 SNVs) association scores. Fewer SNVs (109) showed support from the subsequent association score (score.3), and some SNVs were supported by multiple scores (6 by both score.1 and score.2, 21 by score.1 and score.3, and 2 by score.2 and score.3; Table S1).

**Figure 4:**
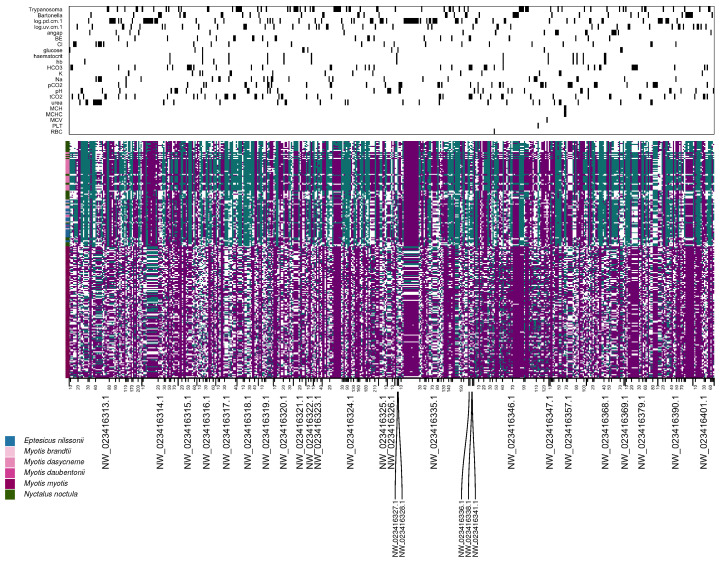
Genotypedistribution and trait associations for 515 SNVs significantly associated with at least one variable in treeWAS analysis. In the genotype matrix, homozygous genotypes polarised towards *Myotis* are shown in purple, and those polarised towards *Nyctalus* in green. White indicates missing or heterozygous genotypes (heterozygotes are excluded by treeWAS). The tick mark colours on the y-axis denote species identity as shown in the legend. The order of the 239 samples is based on the proportion of green alleles and differs from that in Figure 3. The upper panel shows a binary matrix indicating for each SNV (columns) and each variable (rows) whether the association was significant (black) or not (white).

A genotype matrix visualising all significant SNVs across individuals revealed shared polymorphisms between distantly related species and genomic regions enriched for SNVs associated with specific health variables (Figure 4). Infection by *P. destructans* (fungal load) and *Trypanosoma* was associated with clusters of significant SNVs on scaffolds NW_023416314, NW_023416335, and NW_023416346, corresponding to genes *DDX10*, *SFRP1*, and *NEXMIF* (Figure 4, Table S1). Physiological variables such as urea and HCO_3_ showed blocks of significant SNVs on scaffolds NW_023416313, NW_023416315, and NW_023416379, most of which were not located within protein-coding genes, except for *BANK1*.

**Figure 5:**
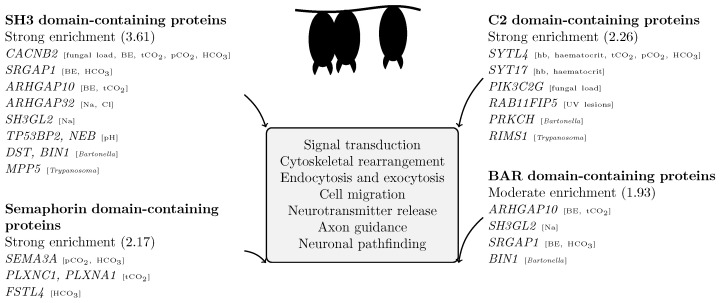
Functional annotation clusters of genes with SNVs significantly associated with any variable, based on DAVID enrichment analysis using *Myotis myotis* as background. Four main protein domain groups emerged with moderate to strong enrichment: SH3, C2, semaphorin, and BAR domain-containing proteins. These domains are involved in cytoskeletal regulation, membrane remodelling, and cell signalling pathways including axon guidance. Gene names are followed in square brackets by the variables for which they showed a significant SNV association in treeWAS analyses. See Table S2 for details.

To explore functional convergence among significant loci, we mapped all significant SNVs to gene annotations and identified 137 unique genes. These were analysed using DAVID functional annotation clustering. We found five annotation clusters with enrichment scores above 1.8, indicating shared functional domains among associated genes (Table S2, Figure 5).

The top cluster (enrichment score 3.61) included SH3 domain-containing proteins. Other clusters with significant enrichment included C2 domains (2.26), Semaphorin domains (2.17), BAR domains (1.93), genes in the KEGG Axon Guidance pathway (1.87), Rho GTPase-activating proteins (1.77), and ATP-binding proteins (1.43). The remaining 17 identified clusters had lower enrichment scores (Table S2).

## 4. Discussion

### 4.1. Infection Tolerance and Physiological Trade-Offs in Hibernating Bats

Studies on tropical bat species indicate tolerance to both *Trypanosoma* and *Bartonella* infections, characterised by high prevalence but minimal clinical or proteomic disturbance, suggesting limited pathology [51,52]. In temperate regions, evidence for infection tolerance during hibernation is strongest for *Trypanosoma*. In hibernating bats, *Trypanosoma* infections showed no measurable effects on blood parameters during torpor [35], and phylogenetic studies confirm widespread but generally non-pathogenic *Trypanosoma* lineages in bat hosts [53,54].

Comparable physiological data for *Bartonella* in temperate, hibernating bats are currently lacking. However, the deep diversification and stable persistence of *Bartonella* across bat lineages imply long-term host–pathogen coadaptation consistent with tolerance rather than acute disease, potentially extending to hibernating taxa [55,56].

Fungal infections provide a parallel example of tolerance under hibernation constraints. Infection by *P. destructans* is widespread in regions of pathogen endemism but largely uncoupled from large-scale mortality or systemic pathology, consistent with a tolerance strategy rather than active resistance [22,57,58]. Although *P. destructans* causes localised wing damage, its haematological and population-level impacts in most Palearctic species are comparatively mild [13,14,15,16,23,32,58], suggesting an evolutionary equilibrium between host and pathogen.

Our study is consistent with the view that temperate bats predominantly tolerate, rather than resist, chronic infections during hibernation [11,12,14,22,23,31,32,57]. The detection of *P. destructans*, *Trypanosoma*, and high *Bartonella* prevalence across multiple species confirms that these hosts carry diverse pathogens often without overt disease. This pattern fits the well-documented suppression of metabolic and immune activity during torpor and suggests that selection favours physiological resilience over active pathogen clearance [3,7,11,59]. In this context, infection tolerance emerges as a by-product of energy conservation strategies that constrain immune activation while permitting long-term pathogen persistence.

Within this tolerance framework, maintaining systemic homeostasis appears critical. The substantial inter-individual variation observed in biochemical and haematological parameters (Table 1) likely reflects differences in hibernation stage, arousal timing, and time elapsed since the last torpor bout, in addition to species-, locality-, and sex-specific effects. Parameters such as HCO_3_, urea, and ion concentrations are sensitive to prolonged torpor and metabolic suppression [25,60,61]. Variation in these traits may therefore influence individual responses to infection while simultaneously serving as indicators of overall physiological robustness. The genome-wide associations detected in this study are thus likely to capture genetic contributions to both infection tolerance per se and the broader regulation of physiological stability under low-energy conditions.

Interestingly, the phylogeny from the whole-genome SNVs (Figure 3) confirms deep genomic divergence among genera, but also between species of the genus *Myotis* c.f. [62]. In contrast, genetic distances within *Myotis* species are minimal and lower than those observed in *Nyctalus* and *Eptesicus*, despite comparable geographic distances between sites with conspecific samples (Figure 1). This is consistent with weak or regionally constrained population structure in several *Myotis* species [63,64], but higher admixture in *N. noctula* [65].

Despite this interspecific separation, many SNVs significantly associated with health-related variables (Figure 4) are shared among distantly related taxa. This suggests that loci influencing physiological and infection-related variation are not lineage-specific but occur in regions of conserved or repeatedly targeted functional diversity. Such overlap may result from ancestral polymorphism maintained across species or from convergent selection on similar pathways under hibernation constraints [66]. The persistence of shared allelic states across divergent lineages thus points to common selective pressures acting on cellular signalling and metabolic regulation that transcend phylogenetic divergence and may contribute to physiological resilience during hibernation.

### 4.2. Functional Convergence of Associated Loci

The 137 genes containing significant SNVs showed convergence on protein domains linked to cytoskeletal organisation, membrane dynamics, and intracellular signalling (Figure 5). The strongest enrichment was observed for SH3 domain-containing proteins, which regulate actin assembly and vesicle trafficking [67]. Other recurrent domains included C2 (calcium-dependent membrane binding), BAR (membrane curvature sensing), and semaphorin domains, together pointing to coordinated control of membrane-associated signalling and cytoskeletal remodelling. These processes are central to cell integrity, endocytosis, and ion transport [68], and thus likely contribute to maintaining homeostasis under the metabolic and osmotic stress of hibernation.

Although associations were detected for diverse variables, including infection and blood chemistry traits, the convergence of functional annotations across all significant loci suggests that selection acts primarily on shared regulatory and signalling mechanisms rather than trait-specific pathways. The enrichment of genes involved in cytoskeletal regulation and membrane trafficking therefore supports a model where tolerance and physiological stability during hibernation rely on conserved cellular processes that integrate immune, neural, and metabolic functions [27,66].

While these associations provide insight into genomic processes underlying physiological variation during hibernation, several limitations should be acknowledged [69]. Sample sizes varied substantially among phenotypic variables due to logistical constraints of field sampling during hibernation, reducing statistical power for some haematological and biochemical traits and necessitating cautious, aggregate-level interpretation of variable-specific associations. Phenotypic data were incomplete across individuals, such that each trait was analysed on a partially overlapping subset of samples, limiting direct comparisons among variables. In addition, individuals were sampled at a single time point per hibernaculum, and no individual-level information on torpor-arousal history was available; consequently, observed phenotypic variation likely reflects a combination of genetic effects and unmeasured physiological state. From a genomic perspective, reliance on a single reference genome may have constrained gene annotation in non-model taxa, and the ddRAD-based variant set captures only a fraction of genome-wide diversity. Finally, although the phylogenetically informed treeWAS framework accounts for shared evolutionary history, it detects statistical associations and cannot distinguish direct functional effects from linked variation. The identified genes and pathways should therefore be interpreted as hypotheses for future validation through transcriptomic or functional assays in hibernating bats.

## 5. Conclusions

Our findings indicate that physiological responses to infection during hibernation are primarily mediated by genes involved in intracellular signalling and energy regulation, rather than canonical immune effector pathways. The significant associations with traits such as fungal load, trypanosome infection, and acid–base balance were concentrated in genes with SH3, C2, BAR, and semaphorin domains, suggesting that membrane dynamics, cytoskeletal rearrangement, and signal integration are key components of overwinter health. These results point to a central role for regulatory and metabolic processes in shaping host–pathogen interactions during torpor, with potential implications for identifying species or individuals at greater risk of emerging infections.

## Data Availability

All data presented in this study are available in the article and its Supplementary Information, and in ENA SRA (accessions: PRJNA681157 and PRJEB104021).

## Supplementary Materials

The following supporting information can be downloaded at https://www.mdpi.com/article/10.3390/pathogens15020149/s1, Table S1: List of all SNVs significantly associated with at least one variable in the hibernating vespertilionid bats based on treeWAS analysis; Table S2: Functional annotation clusters identified from genes with SNVs significantly associated with health-related variables in vespertilionid bats; Figure S1: Boxplots show the median sequencing depth of captured loci (DP > 0) for individual samples from the two independently constructed ddRAD libraries (Library 1 and Library 2), stratified by species.

## Abbreviations

The following abbreviations are used in this manuscript:

angap	Anion gap
BE	Base excess
DAVID	Database for Annotation, Visualization and Integrated Discovery
hb	Haemoglobin
HCO_3_	Bicarbonate
MCH	Mean corpuscular haemoglobin in RBC
MCHC	MCH concentration
MCV	Mean corpuscular volume
pCO_2_	Partial CO_2_ pressure
PLT	Trombocyte count
RBC	Red blood cells
SNV	Single nucleotide variant
SRA	Short-read archive
tCO_2_	Total CO_2_
UV	Ultra-violet
WBC	White blood cells
