# Supplementary material for: Genome-Wide Association Reveals Signalling-Linked Infection Tolerance in Hibernating Bats
Source: Pathogens. 2026 Jan 30;15(2):149. doi: 10.3390/pathogens15020149 (PMC12943343; doi:10.3390/pathogens15020149)
Supplement: Supplementary file 1 [file pathogens-15-00149-s001.zip › FigureS1.pdf]

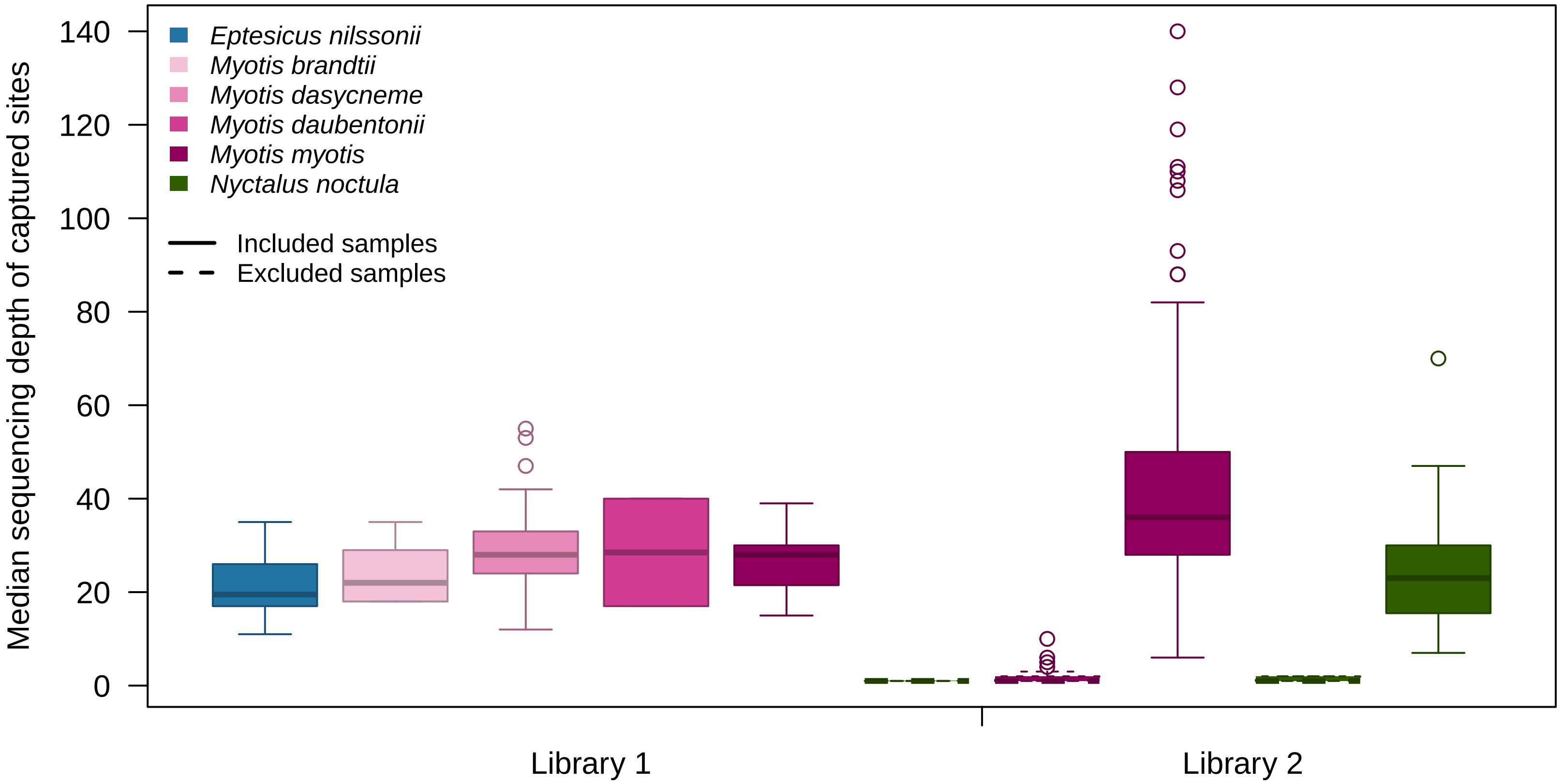
Eptesicus nilssonii
Myotis brandtii
Myotis dasycneme
Myotis daubentonii
Myotis myotis
Nyctalus noctula
Included samples
Excluded samples
Median sequencing depth of captured sites
0
20
40
60
80
100
120
140
Library 1
Library 2
